# Conservation Practitioners’ Perspectives on Management Responses to Poaching in South African Protected Areas

**DOI:** 10.1007/s00267-026-02634-7

**Published:** 2026-10-10

**Authors:** Ndivhuwo Shivambu, Tinyiko Cavin Shivambu, Takalani Nelufule, Tsireledzo Goodwill Makwarela, Tshifhiwa Constance Nangammbi, Nimmi Seoraj-Pillai

**Affiliations:** 1 Department of Nature Conservation, Faculty of Science, Tshwane University of Technology, Pretoria West, 0183 South Africa; 2 College of Agriculture and Environmental Sciences, Department of Environmental Sciences, Florida Science Campus, University of South Africa, Johannesburg, Roodepoort, 1709 South Africa; 3 School of Animal, Plant and Environmental Sciences, University of the Witwatersrand, Johannesburg, South Africa

**Keywords:** Community engagement, Conservation management, Enforcement, Poaching, Protected areas

## Abstract

Biodiversity across Africa faces escalating threats from agricultural expansion, urbanisation, resource overexploitation, and pollution, particularly at the margins of protected areas (PAs). These land‑use changes intensify interactions between people and wildlife by compressing habitats, increasing competition for space and resources, and disrupting ecological processes, thereby exacerbating human–wildlife conflict and threatening conservation outcomes. In this context, conservation practitioners play an important role in managing these conflicts by implementing conservation management interventions. A total of 49 conservation practitioners across KwaZulu‑Natal, Limpopo, and Mpumalanga in South Africa were interviewed using a semi-structured questionnaire to document the implementation of key conservation measures and to examine their association with practitioner-reported poaching occurrence. Most reserves implemented perimeter fencing and shared boundaries with adjacent local communities. Community engagement and environmental education programmes were also widely reported. The proportion of practitioners reporting poaching was highest in KwaZulu‑Natal (84.2%), followed by Limpopo (71%) and Mpumalanga (61.5%). Binary logistic regression revealed that community engagement was significantly associated with lower odds of poaching. Sustaining PAs requires integrated strategies that combine enforcement, community engagement, and adaptive management.

## Introduction

Conservation practitioners, including field rangers and ecologists, play a crucial role in managing the dynamics of human-wildlife interactions (Warchol and Kapla 2012; Kansky, 2022). Beyond enforcing regulations, they are responsible for ecological monitoring, anti‑poaching operations, community engagement, and the day‑to‑day implementation of conservation policies (Milda et al. 2020). As frontline actors, they act as mediators of conflicts and promoters of coexistence between communities and wildlife (Child and Barnes 2010). However, their work is shaped by complex challenges, including limited resources due to financial constraints and political pressures, which significantly influence on-the-ground outcomes (Adeyanju et al. 2021).

At the same time, conservation practitioners must operate within socio‑ecological systems increasingly affected by the expansion of urban and agricultural areas, resource overexploitation, and intensifying environmental pressures (Ban et al. 2013). These factors, as well as increased pollution, continue to pose a significant threat to biodiversity across Africa (Perrings and Halkos 2015; Tilman et al. 2017; Ayeni et al. 2023). These activities are most pronounced at protected areas (PAs) buffer zones, often intensifying land-use competition between communities and wildlife, consequently causing human-wildlife conflict (HWC) (Sinthumule 2014; Baral 2023; Gayo 2025a; Li et al. 2025). To ensure the survival of wild populations and reduce HWC, the role of effectively managed PAs has become essential (Sinthumule 2014; Mengist 2020; Visconti et al. 2019).

The long-term sustainability of PAs is closely tied to the socio‑economic conditions of surrounding communities, especially in developing countries where livelihoods and food security depend heavily on agriculture and natural resources (Thornton et al. 2011; Mengist 2020; Visconti et al. 2019). In response, community-based natural resource management (CBNRM) has emerged as an important approach, fostering local participation in conservation decision-making and ensuring that communities benefit directly from the sustainable use of biodiversity (Ayoo 2007; Lauber et al. 2008; Gibbes and Keys 2010).

Historically, the establishment of conservation areas in Africa has often followed exclusionary conservation principles rooted in colonial ideologies that viewed humans as separate from nature (Singh and Van Houtum 2002; Mutekwa and Gambiza 2017; Domínguez and Luoma 2020; Mthimkhulu and Nel 2024; Gotyi and Handi 2025). As a result, indigenous and local communities were frequently displaced from their ancestral lands, relocated to settlement zones at the edges of PAs, and excluded from from conservation decision-making and resource management (Khan 1994; Carruthers 1995; Anthony 2007; DeGeorges and Reilly 2009; Bro Domínguez and Luoma 2020; Gayo 2025b; Pandey et al. 2025). These circumstances have worsened tensions between PAs managers and communities, mainly due to conflict over damage caused by wildlife and limited access to natural resources such as game meat (e.g., impala, duiker, bushbuck, and kudu), fire-wood and medicinal plants (Brockington 2004; Kepe 2008; Mutanga et al. 2017; Kairiza et al. 2025). The marginalisation of local communities has weakened community stewardship over biodiversity and contributed to reactive practices, such as poaching or the persecution of problem animals (Woodroffe and Frank 2005; DeGeorges and Reilly 2009). These dynamics have highlighted the pressing need to reframe conservation strategies to acknowledge social justice and equitable governance (Nxele et al. 2024).

Through mechanisms such as ecotourism, revenue-sharing, employment, and environmental education, communities are positioned as custodians of nature (Gumede and Nzama 2019; Carius and Job. 2021). Notable examples in South Africa (Oribi Gorge Nature Reserve) and Tanzania (Jozani–Chwaka Bay National Park) demonstrate that such models can support both ecological restoration and rural development (Gumede and Nzama 2019; Carius and Job. 2021). While these initiatives highlight the ability of community-based approaches, understanding the perspectives of those responsible for implementing them remains critical, for example, the role of conservation practitioners. As a result, this study aims to determine the occurrence of practitioner-reported poaching across KwaZulu‑Natal, Limpopo and Mpumalanga. In addition, this study documents the implementation of key conservation management measures across three South African provinces, including fencing infrastructure, staffing capacity, trans‑boundary monitoring, community engagement, and environmental education programmes. We hypothesised that practitioner-reported poaching would differ among the three provinces and that its occurrence would be associated with the implementation of conservation management measures. We further hypothesised that the implementation of conservation management measures would vary among provinces, reflecting differences in conservation management practices across PAs. Understanding these spatial differences is important for identifying management gaps and improving the effectiveness of conservation strategies in human-dominated landscapes.

## Materials and Methods

### Study Area

**Fig. 1:**
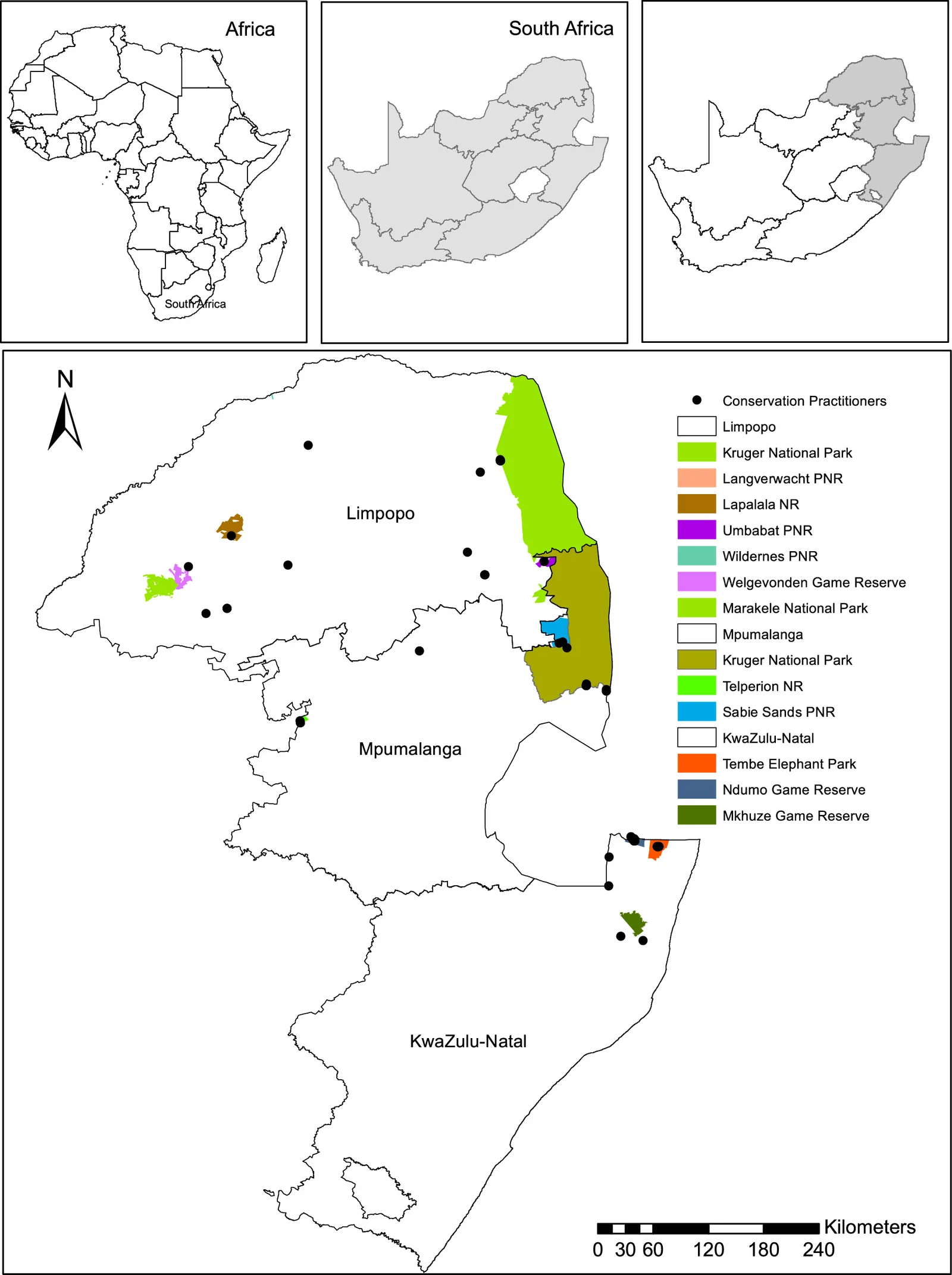
Location of selected protected areas in South Africa where conservation practitioners were surveyed.The map shows their distribution across the provinces of Limpopo, Mpumalanga, and KwaZulu-Natal. Multiple conservation practitioners may be represented by a single point where respondents were employed at the same location; therefore, the number of visible points is lower than the total sample size of 49 respondents

This study was conducted within and around PAs, defined here as formally proclaimed conservation areas under state, provincial, or private ownership, including various game and nature reserves, located across the north-eastern provinces of South Africa, namely KwaZulu-Natal, Limpopo, and Mpumalanga provinces (Fig. 1). These regions were selected due to the significant prevalence of HWC at the interface of agricultural and conservation landscapes (Anthony et al. 2010; Massé 2016; Wentzel et al. 2021; Maseko and Moyo 2022; Makwarela et al. 2026).

### Data Collection

A total of 49 conservation practitioners participated in this study, distributed across KwaZulu-Natal (*n* = 19), Limpopo (*n* = 17), and Mpumalanga (*n *= 13), including field rangers, wildlife ecologists, environmental officers, and game capture personnel (involved in the capture, handling, transport, translocation, and management of wild animals), selected using a stratified random sampling technique. These individuals were employed across diverse conservation entities, including national parks, provincial nature reserves (managed by provincial departments of Economic Development and Tourism), and local authority nature reserves (managed by municipalities). Data were collected through semi-structured questionnaire interviews (Supplementary Material Table S1). The questionnaire administered to conservation practitioners was adapted from a previous survey on farmer-carnivore conflict (Thorn et al. 2012). An open-ended question was also included to prompt conservation practitioners’ opinions on CBNRM. Responses to open-ended questions were presented as illustrative quotes from common explanations.

The anonymity of all respondents was maintained throughout the study. Prior to each survey, a signed consent was obtained from every respondent. Approximately 30-min interviews were conducted at their residences or farms. The 49 respondents were affiliated with 13 PAs, Tembe Elephant Park (2), Ndumo Game Reserve (14), Mkhuze Game Reserve (3) (Kwa-Zulu Natal), Welgevonden Game Reserve (2), Marakele National Park (2), Kruger National Park (6), Langverwacht Nature Reserve (1), Lapalala Wilderness (4), Umbabat Private Nature Reserve (2), Wilderness Private Nature Reserve (2) (Limpopo), Kruger National Park (9), Telperion Nature Reserve (1) and Sabi Sands Private Nature Reserve (1) Mpumalanga. Because Kruger National Park spans both Limpopo and Mpumalanga provinces, respondents were assigned to a province based on the geographic location of the section of the park in which they were employed. In instances where respondents had no English comprehension, a translator was used. Each place of residence or farm served as a single sampling unit and was visited once to minimise pseudo-replication, with interviews restricted to a single respondent per place of residence. Each respondent was associated with a specific protected area (PA) in which they were employed, which served as the primary analytical unit of the study. Place of residence or farms refers to the residences or workplaces of conservation practitioners located within or adjacent to the PAs. Questions that remained unanswered were classified as “no responses”. Demographic information, including language, ethnicity, religion, and formal conservation qualifications, was also collected from respondents via the questionnaire.

### Assessment of Management and Engagement Programmes

The prevalence of transboundary monitoring programmes was assessed among conservation practitioners. These programmes were defined as the surveillance of PA perimeter fences or boundaries designed to prevent wildlife transgressions and mitigate illegal anthropogenic activities such as poaching. Similarly, the implementation of environmental education and community engagement programmes was evaluated. Environmental education programmes were defined as initiatives that inform local communities about the importance of ecosystems and sustainable behaviours, thereby enhancing environmental awareness. Community engagement programmes facilitated meetings between conservation authorities and local communities to foster knowledge exchange, address challenges, and develop solutions collaboratively.

### Statistical Analyses

All statistical analyses were performed using the *R* statistical environment (version 4.5.1; R Core Team 2025). We documented the implementation of key conservation management measures across three provinces, including fencing infrastructure (perimeter and electrified fencing), manpower, transboundary monitoring, community engagement, and environmental education programmes. The implementation of these measures was compared among provinces using generalized linear models (GLMs), followed by post hoc pairwise comparisons where appropriate. Dominant response categories (yes or no) were identified to characterize prevailing management practices. To examine the association between conservation management measures and practitioner-reported poaching occurrence, we fitted a binary logistic regression model with poaching occurrence (yes/no) as the response variable. Explanatory variables included perimeter fencing, electrified fencing, manpower, transboundary monitoring, community engagement programmes, environmental education programmes, and whether the reserve bordered a community. These variables were not quantified as continuous measures (e.g., number of staff, fence length, or programme frequency) but were assessed using binary (yes/no) responses derived from the questionnaire. Model coefficients were interpreted as associations between conservation management measures and reported poaching occurrence rather than evidence of causal relationships.

## Results

**Fig. 2:**
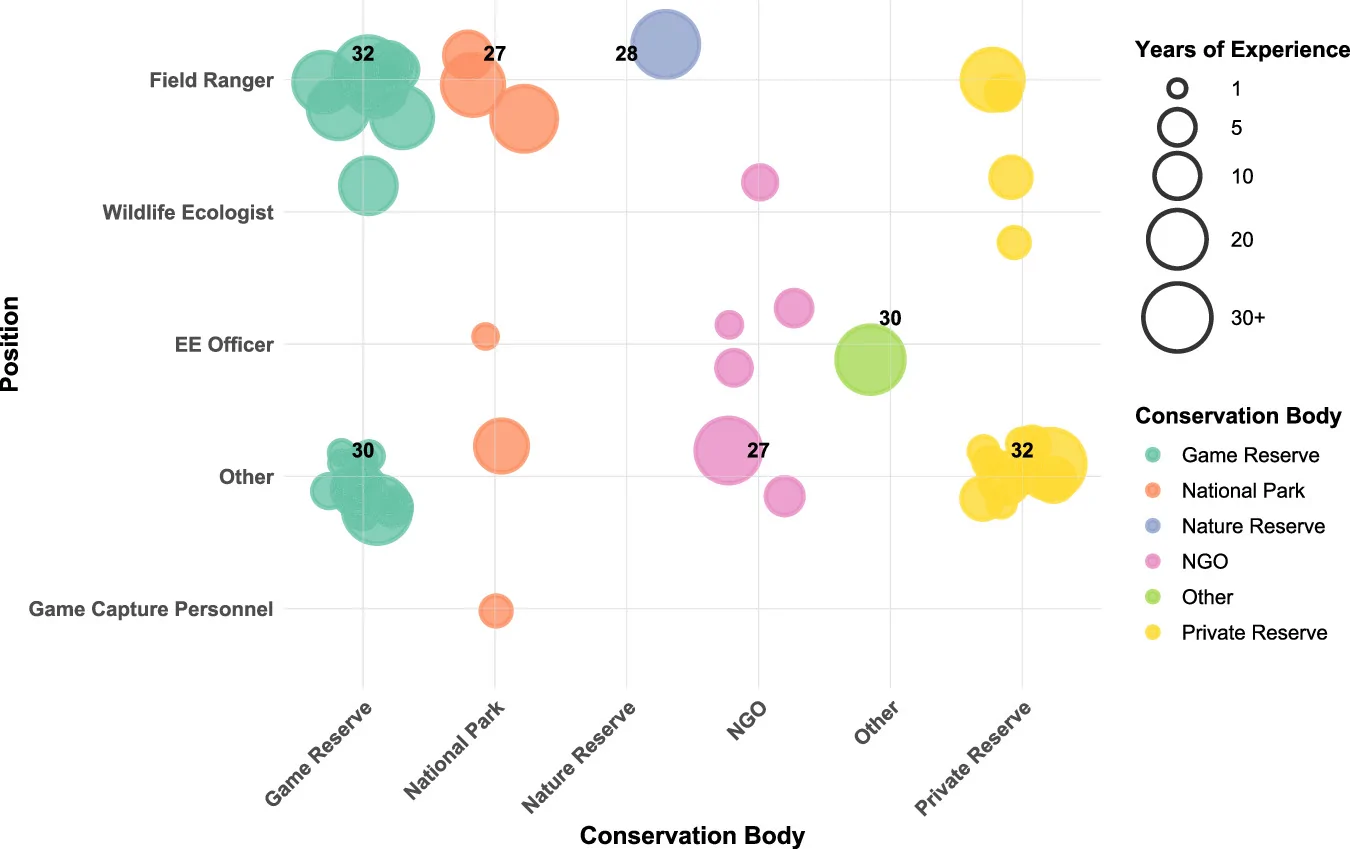
Distribution of positions across different conservation bodies, with bubble size representing years of experience.EE stands for environmental education, while NGO stand for Non-Governmental Organisation

A total of 49 conservation practitioners participated in the study across the provinces of KwaZulu-Natal (*n* = 19), Limpopo (*n* = 17), and Mpumalanga (*n* = 13). Most respondents in KwaZulu-Natal identified as Zulu (57.9%), reflecting the strong representation of local communities within conservation roles in the province. On the other hand, Limpopo and Mpumalanga had higher proportions of white respondents (41.1% and 38.5%, respectively). This suggests differences in historical employment patterns, land ownership structures, and access to conservation training across provinces. Most respondents identified as Christian, and the majority across all provinces held formal qualifications in conservation or a related field (Limpopo: 100%, Mpumalanga: 69%, KwaZulu-Natal: 78.9%), indicating a generally high level of professional training among practitioners. Highly experienced individuals (those with 30 years or more of experience) were concentrated in game reserves and private reserves, particularly among field rangers (Fig. 2). However, field rangers were generally represented across game reserves, national parks, and nature reserves, with some exceeding 30 years of experience, highlighting their central role in on‑the‑ground conservation management regardless of reserve type. Overall, Non‑Governmental Organisations (NGOs) and nature reserves were represented by individuals with less experience (Fig. 2).

**Fig. 3:**
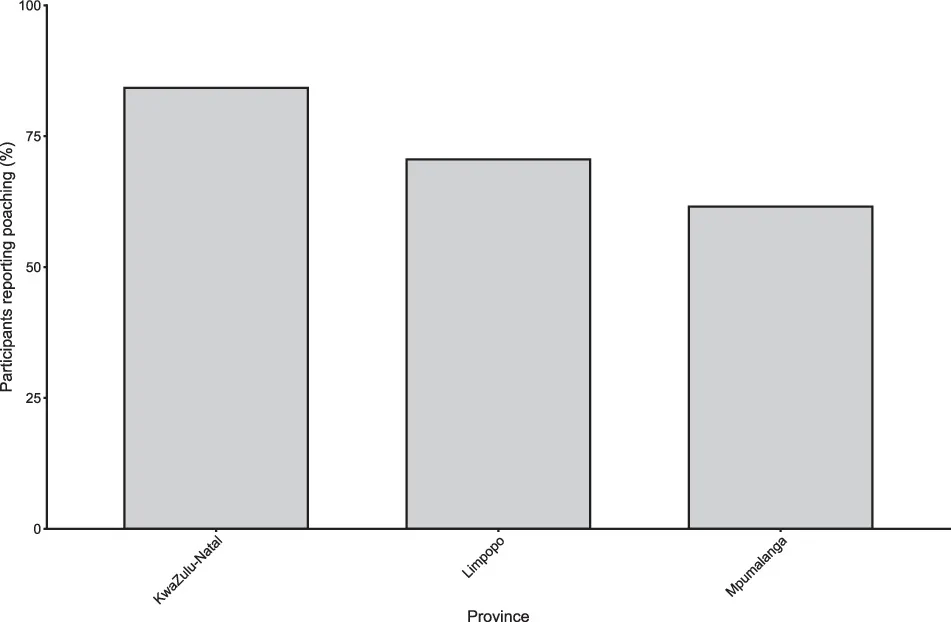
Percentage of participants reporting poaching by province. Bar chart show the proportion of survey participants who reported poaching in KwaZulu-Natal, Limpopo, and Mpumalanga. The y-axis represents the percentage of participants reporting poaching, while the x-axis indicates the study provinces

We found that poaching was the most frequently mentioned anthropogenic problem across provinces. (Fig. 3). The proportion of participants who reported poaching varied across provinces (Fig. 3). In KwaZulu‑Natal, 84.21% of participants (16 out of 19) indicated poaching as a problem. This was followed by Limpopo with 71% (12 out of 17), while Mpumalanga had the lowest proportion at 61.53% (8 out of 13). Although all three provinces showed that most participants were concerned about poaching, KwaZulu‑Natal had the highest relative occurrence (Fig. 3). These findings are consistent with our first hypothesis that practitioner-reported poaching occurrence would vary among the three provinces. Respondents were asked to report whether specific management and mitigation measures were currently implemented at their reserves. These included community engagement programmes, environmental education initiatives, perimeter fencing, and electrified fencing. The majority of conservation practitioners reported implementing perimeter fencing (KwaZulu‑Natal: 100.0%, Limpopo: 88.9%, Mpumalanga: 83.3%), and electrified fencing (KwaZulu‑Natal: 73.7%, Limpopo: 42.1%, Mpumalanga: 38.5%) as well as bordering reserves with community or farm areas (KwaZulu‑Natal: 100.0%, Limpopo: 89.5%, Mpumalanga: 92.3%).

Engagement-related measures were also common, with community engagement programmes (KwaZulu‑Natal: 94.7%, Limpopo: 63.2%, Mpumalanga: 53.8%) and environmental education programmes (KwaZulu‑Natal: 94.7%, Limpopo: 78.9%, Mpumalanga: 84.6%) widely reported. Trans-boundary monitoring was implemented by most respondents (KwaZulu‑Natal: 79%, Limpopo: 65%, Mpumalanga: 92%). In addition, the adequacy of manpower varied across provinces. In KwaZulu-Natal, only 5.3% of respondents indicated sufficient manpower, while 94.7% reported inadequate staffing. Conversely, Limpopo demonstrated a substantially higher capacity, with 68.4% of respondents reporting adequate manpower and 31.6% reporting insufficient manpower. Mpumalanga showed a relatively balanced distribution, with 46.2% of respondents indicating adequate manpower and 53.8% reporting insufficient manpower (Table 1). Overall, these provincial differences support our second hypothesis that the implementation of conservation management measures would vary among provinces.

**Table. 1:** The proportion of conservation practitioners’ responses to the implementation of selected conservation management measures across the three provinces

Variable	Province	Proportion of conservation practitioners by response category		Post‑hoc letters (a and b) indicate statistically significant differences identified through post‑hoc comparisons	Dominant response
		Yes (%)	No (%)	Post-hoc letter	
Perimeter fence	KwaZulu-Natal	100.0	0.0	a	Yes
	Limpopo	88.9	11.1	b	Yes
	Mpumalanga	83.3	16.7	b	Yes
Fence electrified	KwaZulu-Natal	73.7	26.3	a	Yes
	Limpopo	42.1	57.9	b	No
	Mpumalanga	38.5	61.5	b	No
Man Power	KwaZulu-Natal	5.3	94.7	a	No
	Limpopo	68.4	31.6	b	No
	Mpumalanga	46.2	53.8	b	No
Reserve boarder with community	KwaZulu-Natal	100.0	0.0	a	Yes
	Limpopo	89.5	10.5	a	Yes
	Mpumalanga	92.3	7.7	a	Yes
Community engagement	KwaZulu-Natal	94.7	5.3	a	Yes
	Limpopo	63.2	36.8	b	Yes
	Mpumalanga	53.8	46.2	b	Yes
Environmental Education Programme	KwaZulu-Natal	94.7	5.3	a	Yes
	Limpopo	78.9	21.1	a	Yes
	Mpumalanga	84.6	15.4	a	Yes

Values indicate the percentage of respondents showing “Yes” or “No” for each variable

Implementation of conservation management measures was predominantly reported as “yes” responses across variables such as perimeter fencing, reserves bordering community areas, community engagement, and environmental education programmes (Table. 1). We found no significant differences in these variables across provinces; however, significant differences were observed between response categories (‘yes’ versus ‘no’), reflecting strong overall agreement regarding implementation. Conversely, electrified fencing and manpower revealed significant variation between provinces, with KwaZulu‑Natal showing mainly negative responses for manpower, while Limpopo showed a higher proportion of positive responses (Table 1).

We employed a binary logistic regression to investigate the associations between six management variables and the presence of reported poaching. The predictors included perimeter fencing, electrified fencing, manpower, community engagement, environmental education (EE) programme, and whether the reserve bordered a community or farm. Overall, the model was statistically significant (χ² (6) = 15.8, *p* = 0.015) (Table 2). Reserves implementing community engagement programmes exhibited approximately 69% lower odds of reporting poaching than those without (odds ratio [OR] = 0.31, 95% confidence interval [CI]: 0.11–0.87, *p* = 0.025). Electrified fencing was positively associated with poaching (OR = 2.95, 95% CI: 1.12–7.78, *p* = 0.029), indicating that PAs with electrified fencing had higher reported odds of poaching compared to those without. We found no other predictors reaching statistical significance, perimeter fence (OR = 1.23, 95% CI: 0.45–3.35, p = 0.69), manpower (OR = 0.87, 95% CI: 0.32–2.34, *p* = 0.78), EE programme (OR = 0.98, 95% CI: 0.40–2.41, *p* = 0.97), and reserve border with community (OR = 1.45, 95% CI: 0.60–3.48, *p* = 0.41).

**Table. 2:** Binary logistic regression model examining the association between six management measures and the occurrence of poaching (response variable: poaching, yes/no)

Term	Estimate	Std. error	Z-value	*P* value	Odds_ratio	OR_lower	OR_upper
Perimeter fence	0.207	0.512	0.404	0.686	1.230	0.451	3.355
Fence electrified	1.082	0.495	2.186	0.029	2.951	1.118	7.779
Manpower	–0.140	0.505	–0.277	0.782	0.869	0.323	2.339
Community engagement	–1.171	0.523	–2.238	0.025	0.310	0.111	0.865
EE Programme	–0.018	0.458	–0.039	0.969	0.982	0.400	2.410
Reserve boarder with community	0.371	0.447	0.830	0.406	1.449	0.603	3.481

Coefficient estimate (log‑odds), standard error, z‑value, *p* value, odds ratio (exponentiated estimate), and 95% confidence interval for each predictor are shown. Statistical significance is indicated by *p* < 0.05

Regarding CBNRM, respondents shared open-ended views, with most expressing support. In Limpopo, 65% of practitioners were positive, 6% negative, and 29% neutral or gave no response. Mpumalanga showed similar trends, with 62% positive, 23% negative, and 15% neutral. In KwaZulu-Natal, 63% were supportive, 5% opposed, and 32% neutral. Selected comments highlight contrasting perspectives: one practitioner emphasises community awareness, environmental responsibility, and the sustainable use of resources such as grass and muti (traditional medicine), while another criticises subsistence practices, suggesting they risk exploitation under capitalist systems.

## Discussion

Communities residing near PAs can either support or challenge conservation efforts, depending on the balance of costs and benefits associated with their actions (Lotter and Clark 2014; Nana and Tchamadeu 2014; Massé 2020). For example, studies suggest that PAs where local communities perceive limited benefits from conservation may be associated with higher levels of poaching (Massé et al. 2017; Nana and Tchamadeu 2014; Lunstrum and Givá 2020). Conversely, community members are more likely to reduce poaching when they serve as custodians of PAs or participate actively in decision‑making processes, including management and governance activities (Cooney et al. 2017; Massé et al. 2017; Kragt et al. 2020). Most conservation practitioners working in PAs originate from surrounding communities (Elbroch et al. 2011; Haupt 2022). Our study surveyed 49 conservation practitioners, all from the surrounding communities of PAs. Employing conservation practitioners from surrounding communities is important, not only for reducing poaching but also for strengthening conservation initiatives. As a result, game rangers employed by national parks play an important role in maintaining dialogue with residents, encouraging participation in wildlife conservation, and serving as trusted intermediaries between their communities and conservation authorities (Wenborn et al. 2022). However, their strong social ties within local communities can also create ethical and operational challenges, especially when law‑enforcement responsibilities overlap with family ties, friendships, or other social obligations (Van Uhm and Moreto 2018).

Conservation practitioners encounter a range of problems that challenge the effectiveness of their interventions. Our study focused on poaching as it is one of the most problematic issues globally (Lavadinović et al. 2021; Dalpane and Baideldinova 2022). The proportion of conservation practitioners reporting poaching was highest in KwaZulu-Natal, followed by Limpopo and Mpumalanga. These differences may be explained by wildlife value, accessibility, and enforcement capacity. For example, KwaZulu‑Natal has several high‑value rhino populations, which have historically attracted sophisticated poaching networks (Hübschle 2017). Poaching not only threatens conservation initiatives but also directly affects wildlife populations, reducing species numbers, disrupting ecological balance, and compromising the long‑term viability of PAs (Hema et al. 2017; Rija et al. 2020; Moore et al. 2021). This persistent challenge is consistent with studies across Southern Africa that highlight ongoing human–wildlife conflict and illegal resource use (Kahler and Gore 2015; Hiller and Sas-Rolfes 2025). In response to higher poaching rates across regions, conservation managers employ a range of targeted strategies to combat wildlife crime, including installing perimeter and electrified fencing (Dube and Nhamo 2020). Our findings show that perimeter fencing was reported across all three provinces, reflecting its role as a standard baseline measure. However, electrified fencing varied across the provinces, with most respondents recorded in KwaZulu‑Natal. This may indicate that electrified fencing is more commonly implemented in PAs where poaching pressure is perceived to be greater (Lindsey et al. 2012). The positive association between electrified fencing and reported poaching may reflect the tendency to implement electrified fencing in areas where poaching is already a recognised management concern. Because this study did not evaluate changes in poaching over time or the effectiveness of fencing interventions, the result should not be interpreted as evidence that electrified fencing directly influences poaching levels. Rather, it highlights the need for further investigation into the relationship between anti-poaching infrastructure and reported poaching incidents.

Fencing has also been widely used across Africa to mitigate HWC (Makwarela et al. 2026) and reduce poaching, but its success is highly context-dependent and often linked to maintenance costs and ecological trade-offs (Lindsey et al. 2012; Pekor et al. 2019). Spatial considerations also play an important role in reducing poaching. The strategic placement of fencing along boundaries reflects attempts to mitigate edge effects, yet it also signals areas of concentrated risk. Previous studies, which found that poaching incidents in Namibia were significantly clustered near human settlements and access points, emphasise the friction of distance in illegal wildlife offtake (Ferreguetti et al. 2018; Duporge et al. 2020; Nieman and Nieman 2024). Deployment of manpower differed across provinces, with only Limpopo province reporting adequate staffing. Understaffing is an enduring issue across many PAs, and most reserves operate with less than half the recommended ranger density (Rytwinski et al. 2026). In many PAs, underfunding and persistent staff shortages result in patrol teams being sparsely distributed across extensive, often difficult terrain, limiting their capacity to provide consistent spatial coverage and leaving high-risk areas intermittently unprotected (Appleton et al. 2022). As a result, manpower is frequently deployed reactively, concentrating rangers in known hotspots following incidents, which may displace poaching activity rather than reduce its overall incidence (Critchlow et al. 2015). Collectively, these dynamics suggest that manpower is a necessary but insufficient component of anti-poaching efforts and is most effective when integrated into a broader, integrated strategy that combines other components, such as community engagement.

Community engagement and environmental education programmes were widely implemented, with KwaZulu‑Natal showing the highest adoption. This suggests a recognition that high poaching pressure necessitates parallel investment in social interventions. Recent studies demonstrate that community participation in conservation is most effective when linked to benefits, such as employment or resource access, rather than awareness alone (Twyman 2000; Kolinski and Milich 2021). This suggests that education programmes, while valuable, must be embedded within broader livelihood strategies to achieve meaningful reductions in poaching.

The generally positive responses toward CBNRM suggest a potential shift from exclusionary conservation models toward approaches that integrate local communities (Nepal 2002; Adeyanju et al. 2021). Similar findings have been reported in Uganda and Botswana, where tourism revenue-sharing and sustainable resource-use programmes improved relations between communities and PAs management, reduced poaching, and encouraged participation in governance (Archabald and Naughton-Treves 2001; Gaodirelwe et al. 2020). Overall, these findings highlight that CBNRM can play a critical role in enhancing conservation outcomes by fostering trust, shared benefits, and local stewardship, thereby reinforcing the effectiveness and long-term legitimacy of PAs governance.

## Recommendations

To advance a more inclusive and resilient model of conservation, this study suggests several integrated recommendations. Community engagement was associated with lower odds of poaching; as a result, conservation agencies should prioritise expanding and deepening community engagement programmes. These should go beyond awareness‑raising to include tangible benefits (employment, revenue sharing, resource access, co‑management roles), as recommended by recent research on CBNRM effectiveness (Gruber 2010; Measham and Lumbasi 2013). Fencing should be deployed strategically only in high‑risk boundary zones, with regular maintenance and monitoring, and combined with community engagement rather than treated as a standalone solution (Lindsey et al. 2012). In addition, the severe manpower shortages in KwaZulu‑Natal require urgent attention. However, simply increasing ranger numbers is unlikely to reduce poaching effectively unless accompanied by intelligence‑led deployment, professional development, and community partnerships. The role of field practitioners should also be strengthened through expanded training in socio‑ecological literacy, conflict‑sensitive conservation, and participatory governance. Finally, lasting impact will require formalised cross‑sector collaboration, bringing conservation agencies together with municipal, agricultural and development partners to address underlying land‑use tensions and align economic incentives with conservation goals.

## Study Limitations

This study was based on conservation practitioners’ perspectives and assessed practitioner-reported occurrence of poaching using a binary (yes/no) response, rather than the frequency or temporal incidence of poaching events. Consequently, the findings do not quantify the intensity or temporal patterns of poaching across PAs. In addition, temporal records of poaching incidents, as well as socioeconomic, environmental, ecological, and community perception variables, were not collected because they were beyond the scope of the questionnaire. Future studies should integrate incident-based poaching records with ecological, socioeconomic, and community-level data to provide a more comprehensive understanding of the drivers of poaching and the effectiveness of conservation management interventions.

## Conclusions

In conclusion, poaching was reported across all provinces; however, the proportion of conservation practitioners reporting poaching was highest in KwaZulu‑Natal. Our regression analysis demonstrated that community engagement programmes are associated with significantly lower odds of poaching, providing strong quantitative support for community‑based approaches. Conversely, electrified fencing did not independently reduce poaching after accounting for reserve‑level differences, suggesting that its widespread deployment should be re‑evaluated in favour of more targeted, context‑sensitive applications. Difficulties in combating poaching are exacerbated by persistent under‑resourcing, limited manpower, and the need to balance community relations with enforcement responsibilities. Collectively, these impacts highlight the vulnerability of PAs and the necessity for adaptive, inclusive strategies that integrate ecological resilience, community participation, and coordinated governance to sustain conservation efforts.

## Supplementary information

Supplementary Material Table S1

Supplementary Material Table S2

## Data Availability

The data used for this study cannot be publicly made available due to ethical restrictions and participants’ confidentiality. However, the anonymised part of the data can be made available upon reasonable request from the Authors.
