# Supplementary material for: Conservation Practitioners’ Perspectives on Management Responses to Poaching in South African Protected Areas
Source: Environ Manage. 2026 Oct 10;76(10):338. doi: 10.1007/s00267-026-02634-7 (PMC13653863; doi:10.1007/s00267-026-02634-7)
Supplement: Supplementary file 1 [file 267_2026_2634_MOESM1_ESM.docx]

Table S1: Protected areas represented by conservation practitioner respondents included in the study across Limpopo, Mpumalanga, and KwaZulu-Natal provinces.

| **Province** | **Protected Area** | **Management Authority** | **Approximate Size (ha)** | **Conservation Status** |
| --- | --- | --- | --- | --- |
| Limpopo | Kruger National Park (northern section) | South African National Parks (SANParks) | ~1,948,500 | National Park |
| Limpopo | Langverwacht Nature Reserve | Limpopo Tourism and Parks Board | ~4,700 | Provincial Nature Reserve |
| Limpopo | Lapalala Wilderness | Private Conservation Area/Foundation | ~48,000 | Private Nature Reserve |
| Limpopo | Umbabat Private Nature Reserve | Associated Private Nature Reserves (APNR) | ~53,000 | Private Nature Reserve (Greater Kruger) |
| Limpopo | Wilderness (Waterberg area) | Private Conservation Area | Variable | Private Conservation Area |
| Limpopo | Welgevonden Game Reserve | Private Management Authority | ~37,500 | Private Game Reserve |
| Limpopo | Marakele National Park | South African National Parks (SANParks) | ~67,000 | National Park |
| Mpumalanga | Kruger National Park (southern section) | South African National Parks (SANParks) | ~1,948,500 | National Park |
| Mpumalanga | Telperion Nature Reserve | Private Management Authority | ~7,000 | Nature Reserve |
| Mpumalanga | Sabi Sands Private Nature Reserve | Private Management Authority/APNR | ~65,000 | Private Nature Reserve (Greater Kruger) |
| KwaZulu-Natal | Tembe Elephant Park | Ezemvelo KZN Wildlife | ~30,000 | Provincial Protected Area |
| KwaZulu-Natal | Ndumo Game Reserve | Ezemvelo KZN Wildlife | ~10,000 | Provincial Protected Area |
| KwaZulu-Natal | Mkhuze Game Reserve | Ezemvelo KZN Wildlife | ~40,000 | Provincial Protected Area |
