# Supplementary material for: Conservation Practitioners’ Perspectives on Management Responses to Poaching in South African Protected Areas
Source: Environ Manage. 2026 Oct 10;76(10):338. doi: 10.1007/s00267-026-02634-7 (PMC13653863; doi:10.1007/s00267-026-02634-7)
Supplement: Supplementary file 2 [file 267_2026_2634_MOESM2_ESM.docx]

**Supplementary Material Table S1:** The questionnaire survey was designed for 49 conservation practitioners in the northeast region of South Africa.

**CONSENT FORM**

**UNIVERSITY OF WITWATERSRAND**

**SCHOOL OF ANIMAL, PLANT AND ENVIRONMENTAL SCIENCE PhD STUDY QUESTIONNAIRE CONSENT FORM**

**Date : ________________________**

**Questionnaire Number: _________________	Location: __________________________**

Hello, my name is Nimmi Pillai and I am a PhD student at the University of the Witwatersrand in Johannesburg. I would like to invite you to participate in my research project about the interactions between people working in conservation and the communities that border protected areas.

This form is to confirm that you have understood what my study is about and that you are willing to participate in it. Either you can sign your consent yourself at the bottom of the form or I can sign that you have given me permission to proceed with the interview that will take no more than 30 minutes.

**CONSENT**

I hereby agree to participate in the survey study on human-animal conflict mitigation. I understand that I am participating freely and without being forced in any way to do so. I also understand that I can stop this interview at any point should I want to discontinue and that this decision will not in any way affect me negatively.

I understand that this is a research project whose purpose is not necessarily to benefit me personally.

I have received the telephone number of a person to contact should I need to speak about any issues, which may arise in this interview.

I understand that my participation will remain confidential.

I understand that if possible, feedback will be given to my community on the results of the completed research.

____________________	__________________

**Signature of participant	Date**

**Signature of researcher: ___________________________________**

*(This document and the questionnaire will be translated into the 1^st^ language of the participant if required)* **RESPONDENT INFORMATION SHEET- CONSERVATION PRACTITIONER SURVEY**

**UNIVERSITY OF THE WITWATERSRAND**

**SCHOOL OF ANIMAL, PLANT & ENVIRONMENTAL SCIENCES**

**HUMAN-ANIMAL CONFLICT QUESTIONNAIRE SURVEY**

***Information Sheet***

Hello, my name is Nimmi Pillai, a PhD student at Wits University, and I would like to invite you to participate in a questionnaire survey. In advance, thank you for agreeing to take part in this study- your time is appreciated! You have been selected as a potential respondent through a scientific liaison officer at your place of work or due to your position in the field of Wildlife Conservation derived from a Professional database. Your participation in this survey is completely voluntary and if you choose not to take part, you will not be penalised.

The survey forms part of a collaborative study between Wits University, the Endangered Wildlife Trust, the National Research Foundation and Tshwane University of Technology. This study aims to quantify conflict between co-existing subsistence farmers and animals. We are also investigating the experiences of commercial farmers with problem animals. We are interviewing individuals working for Conservation organisations or Game parks to evaluate their attitudes to and experiences with subsistence and commercial farmers. We hope that this survey will benefit rural communities, whilst protecting biodiversity.

The aim of the questionnaire is to gather information about your reserve. The information will help us find out how conservation managers feel about farmers/communities, and allow us to inspect their monitoring programmes, identify conflict hot spots and find ways for conservation managers and communities/ farmers to interact and cooperate much better. Your answers will help find out when, where and how often this conflict happens. We will also use this information to find ways to resolve the problem.

The survey will take about 30 minutes to complete. Questionnaires answered via email can be returned to seorajpillayn@tut.ac.za. Should you wish to anonymously return the questionnaire you may fax it to 086 653 1404. Great effort will be made to keep the information confidential. Contact details are only required so that the research team can give feedback on survey results. In addition, the research team may want information for further research studies to see any changes over time. Contact information will only be shared among members of the research team. If you choose to participate in this survey, you will not be prejudiced in any way. Your responses cannot be associated with your identify. If you feel uncomfortable, at any stage, you may stop and you will not be penalised in any form.

If you have any further questions about the project please feel free to contact my supervisor, Professor Neville Pillay on (011) 717 6459; Neville.Pillay@wits.ac.za or you may call me on 072 2381404. You may also report any complaints to the Human Research Ethics Committee at the University of Witwatersrand, Johannesburg.

Thank you very much for your help and time.

Nimmi Seoraj-Pillai

**QUESTIONNAIRE- CONSERVATION PRACTITIONER SURVEY**

**SURVEY: PEOPLE WORKING IN**

**CONSERVATION**

**Interviewer(s):**___________________________________ **Date:**

**Interviewee:**

1. Title _________ First name ________________ Surname:

***Participant information***

2. Which conservation body do you work for?

- National Park  Game Reserve  Private Reserve  Non-governmental organisation
- Other (please specify) _____________________________________________________

3. What is your occupation? __________________________________________________
4. What is your first language? ______________________________________
5. Postal/Email address:
6. Contact number: ________________________________________________________
7. GPS coordinates: S______________________________ E ______________________
8. How long have you worked in conservation? ______years ______months
9. What tribal group or ethnicity do you identify with? _______________________________  No response
10. What religion do you practice? _______________________________  No response
11. Do you have any formal qualifications related to your position?  Yes  No

 No response

***Reserve attributes***

12. Total size of the site: ____________  **m^2^ /**  **ha** 13. Elevation: _______________ **m**

14. Predominant terrain:  Hilly  Flat  Other ______________________________
15. Does this reserve border a rural community/village/farm?  Yes  No

- 16. If yes, how often do you interact with these people?
- Weekly  Every twee weeks	 Monthly	 Every 6 months
- Once a year	 Once every two years  Other _________________________

17. Does the reserve have a perimeter fence?  Yes  No
18. Is the perimeter fence electrified?  Yes  No
19. What is the predominant biome of the reserve?

- Grassland	 Scrub  Savannah Woodland  Mixed bushveld
- Cultivated fields  Wetland  Other (specify): ______________________

20. Which of the following conditions/problems do you experience on your reserve?

	Low/high rainfall __ Flooding	__ Poor veld condition

	Soil erosion	__ Veld fires	__ Disease/ parasites

	Poaching	__ Tannin/alkaloid toxicity

	Other (specify): ______________________

21. Which antelope species are present on your reserve and in what numbers?

| **Antelope species** | **Numbers** |
| --- | --- |
|  |  |
|  |  |
|  |  |
|  |  |

22. Which of the following species on your reserve?

3085 	Baboon __ African Wild dog	__ Vervet monkey
3086 	Leopard __ Honey badger	__ Cheetah
3087 	Lion	__ Jackal	__ Hyena
3088 	Other (specify): ______________________
3089 23. What is the carrying capacity of the reserve? ____________________________________
3090 24. Is the reserve within its carrying capacity _______________________________________
3091 25. Does the reserve have enough manpower and funds to maintain perimeter fence? 3093  Yes  No
3094 26. How often is your perimeter fences checked for wear and tear?
3095 _________________________________________________________________________ 3096	27. How much money is spent on perimeter fence maintenance?
3097 __________________________________________________________________________________________
3098 ________________________________________________________ 3099	28. Do you implement trans-boundary monitoring at the reserve?  Yes  No 3100	Please provide a reason for your answer.
3101 __________________________________________________________________________________________
3102 ________________________________________________________
3103 ***Interactions with farmers and communities***
3104 29. Do you communicate with farmers bordering your reserve?
3105  Yes  No
3106 30. Do you communicate with rural communities bordering your reserve?
3107  Yes  No
3108 31. If **yes**, how often do you liaise with farmers and rural communities bordering your reserve?
3109 32. Does your reserve have any community engagement programmes implemented currently?
3110  Yes  No
3111 33. If **no**, why? ____________________________________________________________
3112 34. If yes, please give details
3113 __________________________________________________________________________________________

__________________________________________________________________________________________ _______________________________________

35. Does your reserve have any environmental education programmes implemented currently?

 Yes  No

36. If **yes**, why? ____________________________________________________________
37. If **no**, please give details

__________________________________________________________________________________________

__________________________________________________________________________________________ _______________________________________

38. What percentage of local communities is employed at the reserve?

__________________________________________________________________________________________

________________________________________________________

39. What do you think of community-based-natural-resource-management?

__________________________________________________________________________________________

________________________________________________________

40. Do you have a specific animal-damage-control authority at your reserve?  Yes  No
41. If no, how do you deal with human-animal conflict issues?

__________________________________________________________________________________________

__________________________________________________________________________________________ _______________________________________

42. What do you think about the following statements? *(Please tick one that suites you best)*

| **What do you think about the following statements?** | Strongly Agree | Agree | Unsure | Disagree | Strongly Disagree |
| --- | --- | --- | --- | --- | --- |
| Wildlife plays a very important part in our ecosystem |  |  |  |  |  |
| Wildlife attracts ecotourism |  |  |  |  |  |
| Agriculture wastes natural habitats |  |  |  |  |  |
| Poverty is not my problem |  |  |  |  |  |
| Poachers are criminals |  |  |  |  |  |
| Rural communities should benefit from tourism revenue |  |  |  |  |  |
| Educating communities will benefit the reserve |  |  |  |  |  |
| Rural communities can make use of natural resources from/on the reserve |  |  |  |  |  |

***Please return electronic responses to seorajpillayn@tut.ac.za***

**Thank you for your time!**
